# A Low-Cost Hardware/Software Platform for Lossless Real-Time Data Acquisition from Imaging Spectrometers

**DOI:** 10.3390/s23094349

**Published:** 2023-04-28

**Authors:** Jesús Fernández-Conde

**Affiliations:** Signal Theory, Communications, Telematics Systems and Computation Department, Fuenlabrada Engineering School, Rey Juan Carlos University, 28942 Fuenlabrada, Madrid, Spain; jesus.fernandez@urjc.es

**Keywords:** imaging spectrometer, lossless data acquisition, real-time, data storage

## Abstract

In real-time data-intensive applications, achieving real-time data acquisition from sensors and simultaneous storage with the necessary performance is challenging, especially if “no-data-lost” requirements are present. Ad hoc solutions are generally expensive and suffer from a lack of modularity and scalability. In this work, we present a hardware/software platform built using commercial off-the-shelf elements, designed to acquire and store digitized signals captured from imaging spectrometers capable of supporting real-time data acquisition with stringent throughput requirements (sustained rates in the boundaries of 100 MBytes/s) and simultaneous information storage in a lossless fashion. The correct combination of commercial hardware components with a properly configured and optimized multithreaded software application has satisfied the requirements in determinism and capacity for processing and storing large amounts of information in real time, keeping the economic cost of the system low. This real-time data acquisition and storage system has been tested in different conditions and scenarios, being able to successfully capture 100,000 1 Mpx-sized images generated at a nominal speed of 23.5 MHz (input throughput of 94 Mbytes/s, 4 bytes acquired per pixel) and store the corresponding data (300 GBytes of data, 3 bytes stored per pixel) concurrently without any single byte of information lost or altered. The results indicate that, in terms of throughput and storage capacity, the proposed system delivers similar performance to data acquisition systems based on specialized hardware, but at a lower cost, and provides more flexibility and adaptation to changing requirements.

## 1. Introduction

Many emerging data-intensive applications in fields such as aerospace, military, or process control are intended to acquire, transfer, process and store large information volumes at sustained rates of hundreds of Mbps. The main features requested from a hardware/software platform to support real-time data-intensive applications are high global I/O throughput, deterministic temporal behavior, and ample storage capacity.

The achievements of the required time response and I/O throughput without any loss of data are essential issues to address in real-time data acquisition systems. The information must be predictably acquired within precise time intervals, performing in parallel the storage of all the information acquired.

During the last few years, these data acquisition systems have continued demanding higher levels of accuracy and strictness. With increasing frequency, associated requirements force the acquisition and storage of all the digitalized information captured by the input sensors. This implies a significant increase in the required I/O throughput and storage capacity.

Specific systems are generally used to meet this demand. However, they are typically expensive, require ad hoc hardware design and implementation, and require a long time and significant effort to implement and validate. Additionally, these systems are often too specific, needing more modularity to face future changes in system requirements.

This research aims to design, implement and validate a real-time data acquisition and storage system devoted to acquiring and storing images to test imaging spectrometers and associated front-end electronics (FEE). The system is based on commercial off-the-shelf (COTS) hardware elements, incorporating an optimized, multithreaded software application that allows the achievement of the global throughput, temporal determinism, and storage requirements needed in this kind of system, keeping the economic cost of the system and associated development as low as possible. In addition, the system’s overall performance could be improved in parallel with upgrades to any of its integrating components.

The novel content of the presented system is the combination of the following characteristics:

- Only COTS hardware components (no need for specialized hardware);
- General-purpose operating system (no need for a specialized real-time operating system);
- High sustained I/O throughput (100 Mbytes/s approximately);
- Lossless data storage (300 GBytes);
- Optimized and properly configured multithreaded software scheme, using the real-time services provided by the general-purpose operating system.

The demanding acquisition and storage rates needed have imposed challenging constraints on the system architecture. Its COTS hardware components have been pushed to the limits of their performance and capacity, in association with a multithreaded software architecture capable of exploiting the hardware’s full potential.

The rest of the article is organized as follows. Section 2 discusses the background and the main related work. Section 3 addresses the system’s functional description, including the hardware and software architectural schemes. Section 4 is related to evaluating the real-time data acquisition system, including some lessons learned and their application to high-volume real-time data acquisition systems. We will end by summarizing the main conclusions in Section 5.

## 2. Background and Related Work

### 2.1. Background

An increasing variety of aerospace missions exist devoted to providing information and services related to air quality, climate, and the ozone layer [1,2,3,4,5,6,7,8,9,10,11,12].

Sentinel-5P [8], for instance, is a single-instrument spacecraft that carries a push-broom instrument with four hyperspectral channels to identify clouds, aerosols, and atmospheric components. Four spectrometers are included in this instrument, and the instrument uses a push-broom imaging mode to collect spectral data from the ultraviolet to the short-wave infrared with extremely high spatial resolution. This data can be used to determine the local composition of the atmosphere in terms of its gas constituents, clouds, and aerosols. In this fashion, troposphere variability can be studied by combining high spectral resolution, high spatial resolution, and daily global coverage.

The instrument’s acquisition method involves photographing a strip of the Earth with a two-dimensional detector for one second as the satellite moves by about seven kilometers. Using a wide-angle telescope, it can measure a strip the size of 2600 km across the track and 7 km along the track. A new measurement is initiated after a one-second integration, creating a progressive scan of the Earth as the satellite travels. Ground pixels 7 km wide are resolved in the across-track direction and for various wavelengths using the two detector dimensions. The high resolution and small pixel size make it possible to measure natural and artificial sources and sinks of atmospheric gases, including greenhouse gas products.

The five main modules of the instrument are the telescope, relay optics, short-wave infrared spectrometer, instrument control unit, and radiant cooler. The telescope/UVN unit houses the telescope, as well as ultraviolet, visible, and near-infrared spectrometers.

The UVN module houses three spectrometers: UV, UVIS, and NIR. All three spectrometers work on the same concept, passing light from the entrance slit via a collimator lens and grating before each wavelength is detected at a specific location on the detector. The spectrally split picture of the slit is focused onto the 2D detector arrays using a series of camera fore-optics. Similar CCD (charged coupled device) detector arrays with back-illuminated frame transfer technology and split readout registers are used in all three UVN spectrometers.

Capacitive trans-impedance amplifiers convert pixel charge to voltage, and then the signals are routed onto four parallel video output lines for transmission to the FEE. The detector’s four video outputs create corresponding analog output signals read out by the FEE, where analog-to-digital conversion occurs. The FEE also controls and powers the sensor.

More recently, the Sentinel-5 UVNS mission intends to offer daily global coverage with an unprecedented spatial resolution of 7 × 7 km^2^ at Nadir, as compared to heritage instruments such as GOME-2 [13], SCIAMACHY [14] and OMI [15]. The high spatial resolution will enable more accurate detection of emission sources and provide an increased number of cloud-free ground pixels. The spatial resolution is much smaller than in previous missions, imposing demanding requirements on the FEE and the associated testing systems.

These testing systems [16,17,18] need to work under challenging conditions (high I/O throughput, simultaneous data acquisition, and storage, without loss or alteration of data). At the same time, their economic cost must be reduced.

### 2.2. Related Work

Classic real-time data acquisition systems such as [19], even when including expensive hardware components and integrating different real-time operating systems, were limited to a global I/O throughput performance of a few Mbytes/s.

In [20], a real-time data acquisition and processing system for MHz repetition rate image sensors is presented. The system uses modern FPGA circuits to help in the efficient collection and processing of data. The solution is based on Xilinx 7-Series FPGA circuits and implements a custom latency-optimized architecture utilizing the AXI4 family of interfaces. The achievement of theoretical rates of 3.125 Gb/s was expected during the tests. However, the card-to-host DMA engine superimposed the actual performance limit, requiring around 29 ms to complete the transfer (5.12 MB ÷ 29 ms = 176.6 MB/s). The low DMA throughput was caused by the lack of circular buffer support in the ChimeraTK (a PCIe device driver library providing device read/write functionality).

The research work in [21] introduces the design of data acquisition software for a Linear Energy Transfer spectrometer. The software consists of three modules to read out and preliminarily analyze the data: the readout and control module, the data real-time imaging module, and the offline data analysis module. Raw data received by the upper computer are read into the random access memory (RAM) and then stored on the hard disk. Data receiving and processing are carried out simultaneously, being executed in different computer processes. The maximum rate of the data generated by the electronics is below 6 Mbit/s, and there are three layers of electronics. As a result, the data-receiving rate of the upper computer will not exceed 18 Mbit/s. Low data rates make it possible to process data in real time.

In [22], a configuration and control framework for real-time data acquisition systems is presented. It is based on multi-processor system-on-chip (MPSoC), allowing for a customized partitioning of the given workload into software and hardware. Their development primarily targets the application domains concerning the readout of superconducting sensors and quantum bits. Both fields need highly-customized FPGA processing due to performance and latency requirements. The measured data throughput with plain byte arrays results in 117 Mbytes/s speeds.

The system described in [23] allows the measurement of signals with a high degree of resolution. Even with real-time data transmission, the low power consumption permits the systems to run for months only using batteries, allowing installation in difficult access areas. Low power consumption increases autonomous operation and minimizes battery shifting. The low-cost design allows a high-density sampling network, fast maintenance, and substitution. Data rates are in the range of hundreds of Kbytes/s.

In summary, none of the systems considered above integrate all the characteristics needed to acquire and store digitized information at sustained rates in the boundaries of 100 Mbytes/s, with the modularity, flexibility, and low economic cost provided by a hardware/software architecture that integrates COTS components and a multithreaded software application using the services of a general-purpose operating system.

## 3. Real-Time Data Acquisition and Storage System

### 3.1. System Requirements

The set of initial requirements for a data acquisition system devoted to testing spectrometers and FEEs of aerospace missions similar to [8,11] is summarized below:

1. Real-time acquisition and storage of large amounts of information, with timing constraints on the communication with the spectrometer that carries the imaging sensors;
2. Sustained input throughput of 94 Mbytes/s;
3. Storage capacity for 100,000 frames (1 Mpx size per frame, 300 GBytes approximately);
4. No data shall be lost or altered;
5. Low economic cost.

These demanding requirements impose a need to base the system design on heterogeneous hardware/software architectural schemes. Components selected considering their limits regarding I/O throughput, storage capacity, computing performance, and economic cost have been integrated into the final architecture.

### 3.2. Functional Description

**Figure 1:**
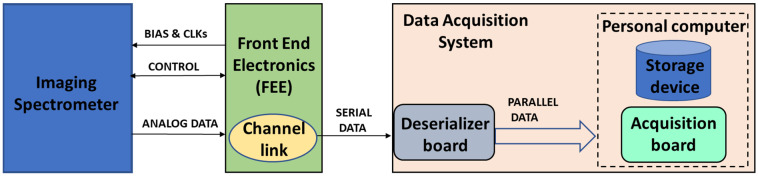
Real-time data acquisition and storage system.

The real-time data acquisition and storage system (RTDAS) presented in this work is depicted in Figure 1.

The front-end electronics (FEE) is responsible for commanding and powering the imaging spectrometer, as well as receiving four different analog output signals provided by the imaging spectrometer (corresponding to four separate detector amplifiers). Inside the FEE, four 14-bit analog-to-digital converters complete digitization, from which science and housekeeping data are relayed to the RTDAS.

Due to a lack of I/O communication pins in the FEE, the data are issued by the FEE in serial format through the channel link. The FEE to RTDAS channel link protocol is based on the emission of 21-bit self-contained words, which have the following formatting:

Bit 20: Data Valid. This bit is set to ‘1’ when the content of the rest of the word is valid. Otherwise, this bit shall be set to ‘0’, and all bits 19-0 shall be set to zero.

Bit 19: Science/Sync. If this bit is set to ‘1’, the payload data represents the science data and originating detector output port, plus a parity bit. If this bit is zero, the payload data represents synchronization data.

Bit 18-0: Payload data in case Science/Sync = ‘1’ (science data):

- Bit 18: Parity (even)
- Bits 17-16: Detector output amplifier (“00” = 1st, “01” = 2nd, “10” = 3rd, “11” = 4th)
- Bits 15-0: Science data

Bit 18-0: Payload data in case Science/Sync = ‘0’ (synchronization):

- Bit 18: Parity (even)
- Bit 17: Frame Synchronization
- Bit 16: Line Synchronization
- Bits 15-14: Data header
- Bit 13: Parity error noticed during previous Frame Period
- Bits 12-0: Spare

The FEE shall send only the following valid words:

- During frame transfer: one sync word with bit “Frame Synchronization” set to ‘1’.
- During each line transfer during readout: one sync word with the bit “Line Synchronization” set to ‘1’.
- At each pixel digitization, the FEE shall send the four pixels in the following order: 1st amplifier, 2nd amplifier, 3rd amplifier, 4th amplifier. For a given amplifier, the data sent to the RTDAS shall follow the order they were read out without buffering or re-ordering.

The FEE can send these 21-bit words at different rates, being a nominal clock equal to 23.5 MHz and a maximum clock rate of 35 MHz. The acquisition system must acquire all the words with bit 20 other than ‘0’ and discard the rest.

The 23.5 MHz nominal clock frequency involves the acquisition of 1 pixel (21 bits of information, packed as a 32-bit word) nominally every 42.55 ns (28.55 ns at maximum clock rate). Consequently, the global I/O throughput demanded from the RTDAS system is nominally 23.5 × 4 = 94 MBytes/s. (maximum 140 Mbytes/s).

The requirement for the number of images to be stored determines the storage capacity needed by the RTDAS system. The storage of 100,000 images with a size of 1024 × 1024 pixels requires over 300 GBytes (only 24 bits per pixel are stored), and no data compression is allowed because the information must be stored without any distortion.

**Figure 2:**
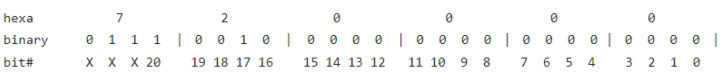
21-bit Frame Synchronization word packed in 3 bytes.

Each 21-bit data word is stored using 24 bits, and consequently, the three most significant bits of each word store do not have meaningful information. For example, Figure 2 presents a Frame Synchronization word (three bytes with values 0x72 0x00 0x00 in hexadecimal format).

**Figure 3:**
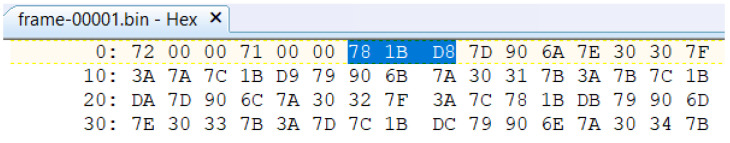
Frame stored using a 3-byte storage scheme per 21-bit data word.

In Figure 3, we can observe the first bytes of a stored frame. The first word is a Frame Synchronization (0x72 0x00 0x00), followed by a Line Synchronization word (0x71 0x00 0x00), and then several science data words. The first science data word (0x78 0x1B 0xD8) can be decoded as follows:

- Data Valid = 1
- Science/Sync = 1
- Parity = 0
- Detector output amplifier = 00 (first detector)
- Science data = 0x1BD8 (16-bit pixel value)

**Figure 4:**
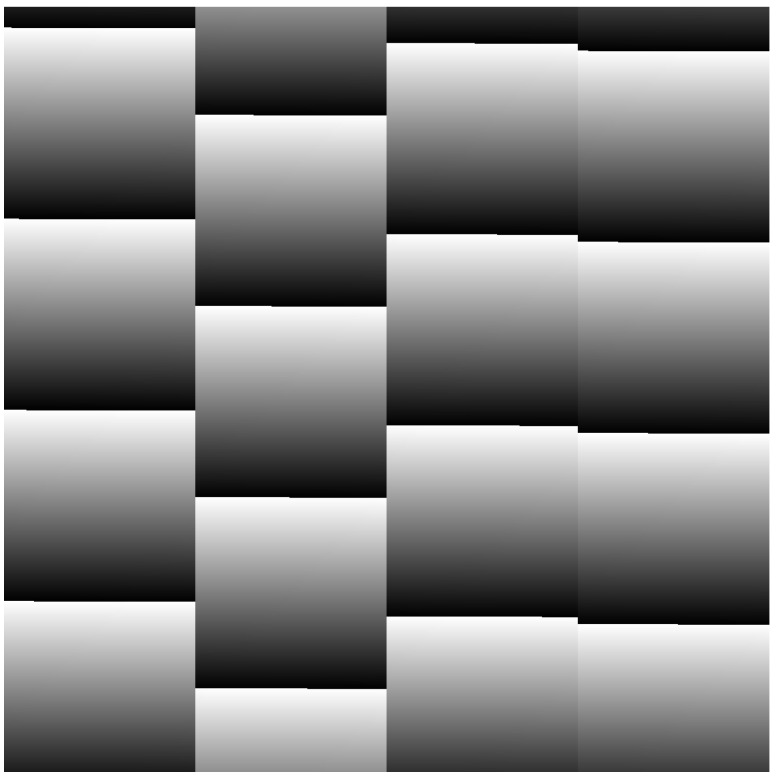
Sample 1 Mpx image, detector amplifiers arranged in four different columns.

Using this storage scheme (3 bytes stored per 21-bit word), the size of a 1 Mpx frame would be equal to 3,148,803 bytes (1 Frame Synchronization, 1024 Line Synchronization, 1024 × 1024 pixels). A sample 1 Mpx image is shown in Figure 4, where we can observe the arrangement of the four different detector amplifiers in four equal-size columns.

### 3.3. Hardware Architecture

#### 3.3.1. Deserializer Board

Due to a lack of I/O communication pins in the FEE, the FEE sends the information in serial format through the channel link. When received by the RTDAS, these data are converted to a 21-bit parallel format on a deserializer board. This activity is carried out transparently, being only necessary to control an extra bit to enable data arrival.

**Figure 5:**
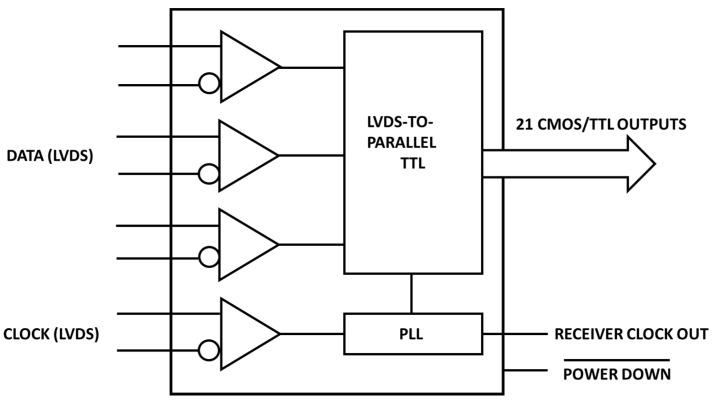
Logical scheme of the Texas Instruments DS90CR218A deserializer board.

The deserializer board used in the RTDAS is the Texas Instruments DS90CR218A [24]. Its receiver deserializes three input LVDS data streams into 21 CMOS/TTL output bits. When operating at the maximum input clock rate of 85 Mhz, the LVDS data are received at 595 Mbps per data channel for a total data throughput of 1.785 Gbit/s (233 Mbytes/s). A logical scheme of the deserializer board is shown in Figure 5.

#### 3.3.2. Acquisition Board

The acquisition board is in charge of acquiring the digitalized information. This digital information is buffered in the acquisition board memory.

The acquisition board used in the RTDAS is ADLINK’s PCIe-7360 [25]. The PCIe-7360 is a high-speed digital I/O board with 32-channel bi-directional parallel I/O lines. Data rates of up to 400 MB/s are available through the ×4 PCI Express interfaces, with clock rates of up to 100 MHz internal clock, ideally suited for high-speed and large-scale digital data acquisition or exchange applications, such as digital image capture. It features 32 channels at up to 100 MHz for digital input, with 400 MB/s maximum throughput.

**Figure 6:**
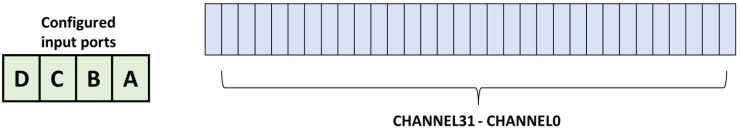
Data mapping for 32-bit data width in ADLINK PCIe-7360 acquisition board.

Thirty-two-channel high-speed digital I/O lines are bi-directional and divided into four groups. Each group contains eight channels and can be configured individually as input or output ports. Data mapping for 32-bit data width is shown in Figure 6.

**Figure 7:**
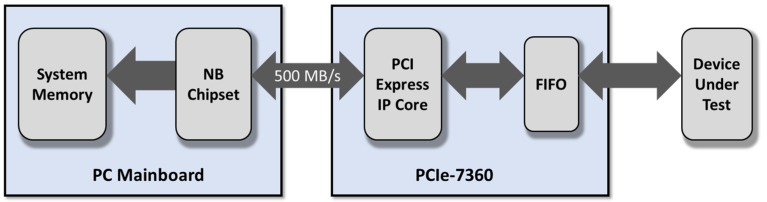
Data transfer between acquisition board and PC memory.

Digital I/O data transfer between PCIe-7360 and PC’s system memory is through bus-mastering DMA, controlled by PCIe IP Core (see Figure 7). Bus-mastering DMA provides the fastest data transfer rate on the PCI/PCIe bus. Once the analog/digital input operation starts, the control is returned to the program. The hardware temporarily stores the acquired data in the onboard Data FIFO and then transfers the data to a user-defined DMA buffer memory in the computer. Data can be transmitted continuously to computer memory (continuous operation).

For the operation of digital pattern acquisition in continuous mode or burst handshake mode, the PCIe-7360 card can acquire digital data from external devices at a specific sampling rate (digital input sample clock). The PCIe-7360 can internally generate the sample clock signal for digital data acquisition, with an internal base clock source of 100 MHz.

#### 3.3.3. Personal Computer

The primary duties assigned to the PC are transferring the data frames from the acquisition board memory buffers to the PC’s local memory and collecting data frames in storage devices.

The PC receives the information from the acquisition board memory, being temporarily constricted in the sense that memory frames have to be read from the buffers located in the acquisition board memory before they fill up completely, i.e., at a higher rate than the filling rate imposed for the sensor and FEE.

The acquisition board manufacturer provides advanced 32/64-bit kernel drivers (DASK) for customized data acquisition application development, enabling complex operations and improved performance and reliability from the data acquisition system. DASK kernel drivers support Windows OS. The board’s memory has a capacity of 2 MBytes, and it can be configured as a different number of buffers, ranging from 2 (double-buffer architecture) to 16.

Using the services of the acquisition board SW driver, the information acquired can be read to the address space of the PC, where it is buffered in local memory and stored in a high-capacity RAID device (and can be visualized offline using a graphical viewer application).

The most important feature required from the storage subsystem is a high-sustained I/O throughput so as not to constitute a global bottleneck. In addition, increased capacity is needed. After considering the utilization of solid-state disks, a RAID disk array was selected as the most suitable storage device, mainly due to the high-sustained data transfer rate and capacity featured. The specific RAID of the final architecture performs over 100 MBytes/s in a sustainable manner, and several hundred GBytes of data can be stored in the array. The characteristics of the storage devices that compose the RAID can be seen in [26].

### 3.4. Software Architecture

The software architecture of the RTDAS has been defined on top of a general-purpose operating system (Windows platform). It comprises several threads which use the real-time resources provided by Windows extensions: automatic memory sharing, parallel execution, real-time scheduling, asynchronous event notification, high-resolution timers, synchronization elements, and multiprocessing. The data acquisition application addressed is appropriate for using a multithreaded scheme, since the system’s performance regarding time response and I/O throughput is highly improved.

The real-time multithreaded process developed for the application, responsible for receiving and storing all the information acquired, is memory resident (virtual address space memory locked) during execution. It is scheduled using a real-time fixed-priority policy.

The main process creates three threads before starting real-time execution: dataReader, diskWriter, and fileManager. During regular operation, these threads run concurrently, sharing data and cooperating to respond adequately to the system’s real-time and I/O throughput demands in a producer–consumer scenario.

The specific functions of the real-time software structure are detailed below, divided into three different stages:

**Stage 1**: Initialization and preparation for the next stage. In this phase, there is only one thread of execution, and real-time requirements are not present. The main functions are to carry out the memory locking of the process virtual address space, initialization of synchronization elements, allocation of memory for buffers, initialization of communication with the Acquisition board through the SW driver, and file opening.

The following system calls and board driver’s functions are used at this stage:

- Register_Card(): open and initialize PCI-7360 card
- DIO_VoltLevelConfig(): set voltage level for DIO ports to 3.3v
- DIO_PortConfig(): configure DIO ports as inputs
- DI_7360_Config(): configure Digital Input (DI) to 32-bit port width, free run mode, no trigger wait, external sample clock
- DI_7360_ExtSampCLKConfig(): set DI sampled clock to source AFI7, Dynamic Delay Adjustment = 0, Dynamic Phase Adjustment = 0
- VirtualAlloc(): allocate multiple buffers to be written, 8-byte alignment; 2 buffers of size 778,240 bytes each
- DI_ContMultiBufferSetup(): set up the allocated buffers; the driver will build the memory descriptor for the specified buffers to perform direct memory access (DMA) operation. The buffers set by DI_ContMultiBufferSetup() are chained to be a ring buffer for the DMA engine to write data to the set buffers continuously.
- fopen(): create/open 500 files for writing.

**Stage 2**: Real-time execution. Real-time requirements are considered in this phase. The main steps are outlined next: creating real-time threads; assigning them real-time priorities; initialization stage of the threads created; and implementation of the acquisition loop (threads resynchronization and starting of concurrent execution).

The following system calls are used at this stage:

- CreateThread(): creation of threads
- SetPriorityClass(): REALTIME_PRIORITY_CLASS
- SetThreadPriority(): assignment of priorities

During the acquisition loop, the real-time software is responsible (among other tasks) for preventing the overflow of acquisition board memory buffers.

**Figure 8:**
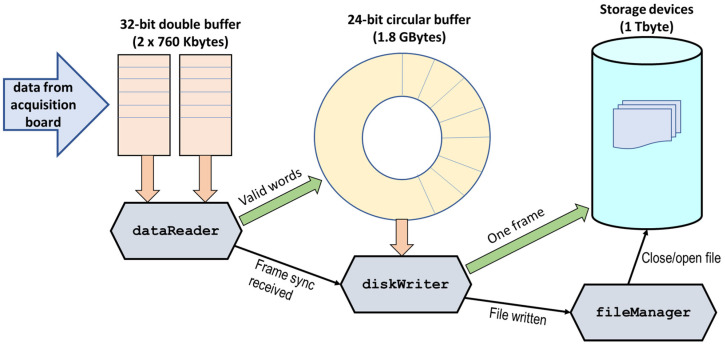
Interaction among the different threads of the RTDAS software architecture.

The different real-time threads which configure the core of the RTDAS software architecture are listed and described below in real-time priority order (from highest to lowest). A diagram showing the interaction among the different threads can be found in Figure 8.

- Thread dataReader: (top priority: TIME_CRITICAL). This thread checks if the next buffer in the ring is filled with data, and consequently, it must be read to avoid data loss. Whenever a buffer is ready to be read, it performs the following actions to process the data:
  1. Reads every 32-bit word in the buffer. For each word in which the DATA_VALID bit is set:
    - Copies the three least significant bytes of the 32-bit word to a local array (treated as a large circular buffer with a size of 1.8 Gbytes).
    - If a 32-bit word is a FRAME_SYNC, it sends a message to diskWriter, because the information buffered in the local array up to that moment is enough to be written to the storage device efficiently.
  2. Tells the driver that the ready buffers are handled entirely (DI_AsyncMultiBuffersHandled())
  3. Checks whether DI is overrun (DI_AsyncDblBufferOverrun())
- Thread diskWriter: This thread waits for messages from dataReader. Whenever a message is received, it writes the blocks of data specified in it to the RAID storage device. The data are read from the local array. A block size of one frame is used (2 Mbytes if the frame has an image size of 1 Mpx.). Every frame is written into a different file. Once a frame is written to disk, it signals the fileManager thread in order to inform it.
- fileManager: This thread waits to be signaled by diskWriter. Whenever it is signaled, it closes the file just written by diskWriter. If the number of files closed is above 490 (meaning less than 10 files remain open), it opens a new file.

**Stage 3**: Conclusion and resource deallocation. In this phase, the following actions are performed: threads termination, the transmission of disconnection order to the Acquisition board, resource deallocation (memory, synchronization elements), unblocking process virtual address space, and closing of all open files.

The following system calls and board driver’s functions are used at this stage:

- CloseHandle(): termination of threads
- Release_Card(): disconnect from PCI-7360 card
- VirtualFree(): memory deallocation
- fclose(): closing of all open files

## 4. Evaluation and Results

The real-time data acquisition hardware/software architecture described in the previous section intends to integrate diverse COTS elements at their full potential regarding performance and capacity to obtain a high-performance, cost-effective data acquisition system.

The modular design of the system guarantees to provide enhanced flexibility and scalability. The heterogeneous components included in its architecture can be replaced flexibly, surpassing the system’s global bottleneck in parallel with future technological innovation in real-time software architectures, high-performance buses, storage devices, or high-speed links.

A significant concern of this type of real-time application is the correct identification of the potential technological bottlenecks that may limit the system’s global I/O throughput. According to the components selected for the RTDAS system, the following theoretical hardware limits have been detected:

- Acquisition board: theoretical 400 MBytes/s data transfer rates can be achieved at full transmission speed, using a 32-bit data width and a 100 MHz clock rate. The interface with the personal computer is PCI Express ×4 (4 GB/s bandwidth, far above the board data throughput limit). The probability of data transmission failure is negligible.
- Deserializer board: according to the manufacturer, 233 Mbytes/s data rates are reachable when operating at maximum clock speed.
- Storage devices: The storage devices used in RTDAS are 1 TB Seagate Barracuda 3.5-inch 7200 rpm HDD. According to the devices’ datasheet, their theoretical sustained writing rate is 210 Mbytes/s. The effective sequential write speed tested in a real benchmark [27] is 193 Mbytes/s. It is worth mentioning that a larger disk capacity implied a lower write speed (for example, a 6 TB disk possesses a theoretically sustained writing rate of 185 Mbytes/s).

Therefore, the theoretical hardware bottleneck of the RTDAS architecture is 193 Mbytes/s. In order for the system to meet the initial specifications (94 Mbytes/s input throughput, simultaneous storage of 1 Mpx 100,000 frames, no data lost or altered) and be as close as possible to reach its theoretical performance limits, some important software design decisions were taken. These options can be outlined as follows:

1. Configuration of the number of buffers in the acquisition board: several configurations of buffers were tested; the best performance was obtained when two buffers were selected (the possible range being from two to sixteen buffers). It is worth noting that the total amount of buffer memory provided by the board drivers is approximately 2 Mbytes, regardless of the number of buffers selected.
2. Multithreaded software scheme: parallel thread execution improves performance, being particularly suited for data-intensive applications that are I/O-limited.
3. Real-time features provided by the operating system: the maximum size of each acquisition board buffer is 778,240 bytes, which makes it fill up every eight milliseconds at a nominal clock speed of 23.5 MHz. This is a tight time for a multitasking time-sharing OS like Windows. Consequently, threads have to be scheduled using a fixed priority scheme.
4. Opening of files: as the maximum number of open files is 512 in Windows OS, it was decided to open 500 files prior to the beginning of data acquisition. In this fashion, during data acquisition, a new file is opened only when a previous file is filled with data and closed.

Finally, after successfully implementing all of the above alternatives, the actual system performance obtained in extensive laboratory testing is summarized in Table 1.

Visualization of a sequence of different images containing test data can be seen in the following video: https://www.loom.com/share/af27824dd09a46e4aecf4001b5790135 (accessed on 17 April 2023).

## 5. Conclusions

In this article, we have covered the description and evaluation of a real-time hardware/software architecture implemented and evaluated to build a data acquisition system needed to perform under severe time constraints and high throughput requirements.

The architecture is heterogeneous and scalable. It includes a data acquisition board and a multithreaded software architecture capable of acquiring and storing data simultaneously at high throughput (94 Mbytes/s), preserving the integrity of 300 GBytes of data at 100%.

The correct combination of COTS components with an optimized and properly configured multithreaded software application running on top of a general-purpose operating system has made possible the satisfaction of the requirements in determinism and capacity for processing and storing large amounts of information in real time, keeping the economic cost of the system low.

Finally, we have also reported the evaluation results of the data acquisition system. We have pointed out the lessons learned when experimentally identifying bottlenecks and dealing with the real-time behavior of the hardware/software architecture. The lessons learned during the analysis and resolution of the problems can surely help future aerospace and embedded software engineering projects.

The system’s modularity and low economic cost will provide future enhanced overall performance and behavior in parallel with technological advances in the different components of the architecture.

## Figures and Tables

**Table 1:** RTDAS system performance.

Clock Speed	23.5 MHz (Nominal)	33 MHz	50 MHz
I/O acquisition throughput	94 Mbytes/s	132 Mbytes/s	200 Mbytes/s
I/O storage throughput	70.5 Mbytes/s	99 Mbytes/s	150 Mbytes/s
Frames lost/altered	None	None	0.01%

## Data Availability

Not applicable.
